# The Impact of Mobile Payment on Household Poverty Vulnerability: A Study Based on CHFS2017 in China

**DOI:** 10.3390/ijerph192114001

**Published:** 2022-10-27

**Authors:** Yuhua Li, Xiheng Gong, Jingyi Zhang, Ziwei Xiang, Chengjun Liao

**Affiliations:** School of Economics and Management, Shanghai Institute of Technology, Shanghai 201418, China

**Keywords:** mobile payment, household poverty vulnerability, entrepreneurship, risk management capability, IVprobit model, electronic communication technology, inclusive finance

## Abstract

Changes in digital technology have brought about new opportunities in the field of financial poverty alleviation in China, and mobile payment as a new digital financial model is important in helping families to lift themselves out of poverty effectively and prevent a return to poverty. This paper examines the impact of mobile payment on household poverty vulnerability and the mechanism of action using the China Household Finance Survey (CHFS) 2017 microsurvey data. After adopting the IVprobit model and a series of robustness tests, we found: (1) mobile payment significantly negatively impacts household poverty vulnerability; (2) the mechanism analysis indicates that promoting entrepreneurship and improving risk management capabilities are the main channels through which mobile payment mitigates household poverty vulnerability; (3) household entrepreneurship and entrepreneurial survival significantly reduce the probability of poverty vulnerability; and (4) the probit regression analysis explores how mobile payment has a greater negative impact on poverty vulnerability among low-income, homeless, and relatively backward households in rural or western areas. This work contributes to the literature on the use of electronic communication technology to eradicate poverty and on inclusive finance, providing vital results for other countries to use as an example.

## 1. Introduction

Poverty eradication is a common vision for the development of human society and the primary goal of the United Nations 2030 Agenda for Sustainable Development. As the largest developing country, China has historically addressed the issue of absolute poverty and achieved the poverty reduction targets of the UN 2030 Agenda for Sustainable Development 10 years ahead of schedule, a feat that has attracted worldwide attention. However, this does not mean the end of the fight against poverty in China. The 2022 Central Government Document No. 1 states that in the face of the 100-year change and the epidemic of the century, the bottom line of no return to poverty on a large scale should be firmly guarded [1]. Some figures show that nearly 2 million people who have escaped poverty are at risk of returning to poverty, and nearly 3 million more people in marginalized groups with high vulnerability are at risk of becoming poor [2]. Therefore, resolutely winning the battle against poverty, consolidating the results of poverty eradication, and building a long-term mechanism to reduce the vulnerability of marginal groups to poverty have become issues that China needs to address urgently.

Financial poverty alleviation is both a major innovation and an important support guarantee in China’s fight against poverty. Chinese President Xi Jinping has repeatedly emphasized the need to do a good job in the article on financial poverty alleviation. It has been pointed out that financial development can alleviate poverty directly through the provision of financial services, such as the provision of credit support, insurance coverage, and other services that play a key role [3,4,5,6,7]. Among them, inclusive finance, as an important initiative of China’s financial reform, has become an indispensable part of financial poverty alleviation by providing sustainable and effective financial services for all social classes and groups. However, the traditional financial system has problems such as asymmetric market information and a high cost of financing for enterprises, resulting in a serious lack of financial “inclusive” services and inhibiting the effect of financial development on poverty reduction [8,9,10,11,12].

Over the past decade, information and communication technologies (ICTs) such as mobile phones and the Internet have been closely integrated with financial services, giving rise to the development of new forms of digital finance, including digital payments, online credit, and smart investment advice. This offers new solutions for promoting financial inclusion [13]. In this area, many have shown that ICT can contribute to the development of financial inclusion [14,15]. For this reason, many researchers have taken a keen interest in exploring the socioeconomic benefits of digital finance. However, much of the current literature has mostly focused on the impact of digital finance on macroeconomics as well as climate change [16,17,18]. In contrast, there is a significant lack of research on the impact of financial technologies on household or individual welfare, particularly on household poverty. 

Currently, mobile payments, an emerging financial technology, have become an integral part of people’s daily lives. According to data from Ariadne Consulting, the total scale of mobile payments and internet payments in China reached RMB 271 trillion in 2020 and is expected to reach RMB 489.2 trillion in 2025, making it the leading international payment market [9]. Mobile payment platforms can provide credit, savings, wealth management, collection, investment, insurance, and other financial services, especially microcredit, effectively reduce the cost of financial services, expand the number of beneficiaries of financial services, and to a certain extent, play a role in financial relief [19,20,21,22,23]. For example, with the credit points generated by mobile payments, low-income groups can obtain small loans, such as Ant credit pay and Ants by chanting, which are simple to review and do not require collateral, helping the poor and vulnerable groups in the process of poverty alleviation, and thus, effectively alleviating poverty [6,7,24,25]. Therefore, can mobile payments, developed through digital technologies such as the Internet and big data, be a “bridge” for the implementation of inclusive financial policies in China? Can they effectively consolidate poverty eradication by alleviating the vulnerability of households to poverty and, thus, help to establish effective mechanisms to prevent the return to poverty?

This paper seeks to explore whether mobile payments can reduce household poverty vulnerability and the mechanisms by which they do so in China. Based on data from the 2017 China Household Finance Survey (CHFS), we conducted several empirical tests and drew useful conclusions. First, we conducted a regression analysis using the probit model and found that mobile payments can significantly reduce household poverty vulnerability. Secondly, we addressed the endogeneity of the probit model using the instrumental variable (IV) approach. As additionally, we performed robustness tests using methods such as replacing the estimated model and reconstructing poverty vulnerability. Our results are consistent with the baseline regressions. Third, we explored the moderating effect of mobile payments. The results show that mobile payments reduce household poverty vulnerability. Fourth, we examined two possible channels through which mobile payments work. Our empirical results suggest that mobile payments reduce household poverty vulnerability through the channels of promoting household entrepreneurship, as well as improving household risk management capabilities. Finally, the results of the heterogeneity analysis suggest that mobile payments have a greater negative impact on poverty vulnerability for low-income, homeless, poor, and vulnerable groups, and households located in rural or relatively disadvantaged areas in the west.

The marginal contribution of this study is twofold: on the one hand, it enriches the research related to digital finance, especially mobile payments, on household poverty vulnerability. Unlike the existing literature which focuses on the impact of mobile payments on innovation and entrepreneurship, investment, and consumption, this study examines the impact relationship and the underlying mechanisms between mobile payments and household poverty from a unique perspective of poverty vulnerability, extending the relevant research on mobile payment. On the other hand, this paper argues that mobile payments can drive the financial inclusion of poor and vulnerable groups and those in less developed regions, providing important policy recommendations for governments to use ICTs to help alleviate people’s hardships and build a long-term mechanism to prevent a return to poverty. This provides some policy insights for other developing countries to improve people’s living standards through mobile payment.

The rest of this paper is arranged as follows: Section 2 introduces the related literature and research hypothesis. Section 3 presents the data, models, and relevant variable descriptions. The empirical results are shown in Section 4. Finally, Section 5 presents the conclusion.

## 2. Literature Review and Research Hypothesis

### 2.1. Literature Review

Vulnerability first originated in the field of natural disasters, where it reflects the degree to which a society is threatened and harmed by natural hazards [26,27,28,29]. The concept of vulnerability to poverty was formally introduced by the World Bank in the World Development Report in 2000 and is defined as the probability that a shock will cause a decline in welfare in the future, i.e., the probability of poverty occurring in future periods. It is an “ex-ante measure” that provides a dynamic and accurate picture of the quality of poverty reduction and the welfare of households. The key to antipoverty policy formulation is how vulnerability to poverty is measured to effectively identify the targets of pro-poor policy attention [30,31,32,33]. The existing literature focuses on measuring poverty vulnerability in three ways: VEU (vulnerability as low expected utility), VEP (vulnerability as expected poverty), and VER (vulnerability as uninsured exposure to risk) [34,35,36,37]. The VEP approach measures poverty vulnerability in terms of dynamic poverty. The VEP approach predicts trends in poverty change from a dynamic poverty perspective, and it is applied to cross-sectional data so that the predictions are forward-looking [32,38]. Its basic logic is that an expression for future income is obtained by regressing the income on observable variables and shocks. It also calculates poverty vulnerability by assuming that future income follows a normal distribution and defining the probability that it will fall below the poverty line as the vulnerability line. It has been found that the use of consumption as an indicator of poverty vulnerability is more robust than that of income [39]. For example, people may use their savings to borrow from others, invest, or buy insurance in the future to smooth out changes in household income from period to period; thus, using consumption levels as an indicator of poverty vulnerability not only reflects changes in income, but also highlights the ability of households to consume. At the same time, depending on the subject of study, some scholars have also used assets as an indicator to measure the probability of falling into poverty in the future, which has the advantage over income indicators of being able to predict a household’s durable and stable income in the future through the various types of assets that can be observed in the current period [40,41].

Thus, what kinds of households are poor and what are the measures of household vulnerability to poverty? The existing literature measures poverty in two main ways. On the one hand, the incidence of poverty is calculated based on a poverty line to classify the poor population. China has been using the rural income poverty line set by the National Bureau of Statistics since the early 1980s. The current standard is a net income of 2300 yuan per person per year that was set in 2010 as a benchmark to calculate the poverty line, and it has been used as the standard for absolute poverty to this day [42]. In the same year, the World Bank set an international consumption poverty line based on purchasing power parity (PPP) calculations of USD 1.9 per person per day, USD 3.1 per person per day in developing countries, and USD 5.5 per person per day in developed countries as the standard. On the other hand, some scholars argue that we should not only consider economic poverty, but also construct multidimensional poverty indicators to measure poverty comprehensively from various aspects such as education, health, and quality of life, thus deepening the identification of poverty [43]. In addition, to accurately measure the vulnerability of households to poverty, Pritchett et al. [44] concluded that a household is vulnerable when the probability of future poverty is greater than a certain threshold value, which was set at 0.5 by Klasen and Waibel [45] depending on the macroeconomic environment, local resources, and human capital of the study population. If the probability of future poverty is greater than 50%, then the household is vulnerable and its prediction accuracy is higher than that of vulnerability found using poverty incidence. However, it has also been argued that converting 50% to a 29% threshold using a time horizon avoids missing households that are temporarily poor and provides a higher level of accuracy [46].

The literature review related to our article has two aspects. The first aspect is the impact of mobile payment on family welfare and the second is the impact of digital finance on poverty. It can be seen from Table 1 that the research on mobile payments only focuses on family welfare, such as income, health risk, and consumption, and does not go deep into the research on poverty. However, the research on vulnerability and digital finance development is limited to data availability, and the impact and mechanism of digital finance on poverty vulnerability have not been explored in depth. This is the first time, in this paper, that the relationship between mobile payment and family poverty vulnerability from the perspective of dynamic poverty is studied, which opens a new perspective for research in related fields.

### 2.2. Research Hypothesis

**Hypothesis** **H1.** *Mobile payment positively influences household poverty vulnerability in China.*

No direct evidence has been given in the literature that new payment methods can reduce poverty vulnerability, but scholars have argued that digital financial services such as mobile payments can have an important impact on the real economy. Lin et al. [55] argue that payments through consumer vouchers can increase national income and have had an expansionary effect on the macroeconomy during the COVID-19 epidemic, thereby boosting mass consumption. Zhang et al. [56] found that mobile payments can be used as a vehicle for digital finance to promote economic growth and alleviate income inequality by alleviating liquidity constraints and facilitating payment channels for residents. In addition, with the rapid development of third-party payments and the continuous improvement of the digital infrastructure in China, digital means such as mobile payments have broken through the geographical constraints of traditional financial poverty alleviation, providing equalized development opportunities for poor and vulnerable groups in different regions and helping them to escape poverty and improve their lives.

**Hypothesis** **H2.** *Mobile payment alleviates household poverty vulnerability through the channel of promoting entrepreneurship in China.*

Entrepreneurship not only contributes to economic growth and income distribution, but also contributes to robust poverty eradication and poverty reduction, effectively reducing poverty vulnerability. The current literature on entrepreneurship for poverty reduction focuses on the following two scenarios. First, Sutter et al. [57] argue that poverty is caused by the lack of resources and capabilities of the poor and that entrepreneurship can compensate for this lack, thus leading to poverty alleviation. For this reason, many studies have attempted to identify solutions that correspond to the “capability poverty” dimension to help grassroots entrepreneurs alleviate poverty through entrepreneurship. Secondly, Williams et al. [58] attribute poverty to the constraints of the local policy system and geographical location. Local poor residents can make full use of the linkage effect between local community entrepreneurship and national/regional precision poverty alleviation policies according to local customs and cultural habits to achieve entrepreneurship for poverty alleviation. At the same time, mobile payments can significantly increase the probability of household entrepreneurship by easing credit constraints and reducing the cost of starting a business, and the use of new payment methods can create more favourable conditions for households to start their businesses, thus increasing their motivation to do so.

**Hypothesis** **H3.** *Mobile payment alleviates household poverty vulnerability through a channel of enhancing the risk management capacity of households in China.*

From the perspective of household economic vulnerability, the main source of vulnerability is risk shocks, which makes the accurate identification and improvement of one’s own risk management capacity a key priority in mitigating poverty vulnerability. In contrast to China, Western households not only use precautionary savings to improve their internal risk management capabilities, but also use financial instruments such as insurance, formal credit, and mature social security systems to hedge against risk. Wang and Kan [59] argue that the emergence of third-party payments has provided an important sales channel for insurance. Compared to traditional insurance products, the use of mobile payments for Internet insurance sales has significantly increased insurance turnover and improved insurance claim rates. In addition, mobile payments help broaden households’ external financing channels and ease their liquidity constraints to protect against external risks. Wang [60] found that mobile payments, mainly through WeChat and Alipay, can be used to analyse big data and the credit ratings of different users’ creditworthiness through online consumer platforms and social media. Moreover, the speed of disbursement and the wide coverage of such microfinance services have greatly lowered the threshold of financial services, bringing new opportunities for poor and vulnerable groups to alleviate their vulnerability. 

## 3. Materials and Methods

### 3.1. Data Source

This article uses data from the China Household Finance Survey (CHFS) conducted nationwide by the China Household Finance Survey and the Research Centre of Southwestern University of Finance and Economics in 2017. The survey has good representativeness as its data on various aspects, such as household assets, liabilities, and demographic structure, are consistent with the data from the National Bureau of Statistics. The CHFS baseline survey was started in 2009 and is conducted every two years, with a nationwide random sample of households having been successfully surveyed four times in 2011, 2013, 2015, and 2017. Currently, the CHFS has become one of the mainstream databases for research on China [61,62]. The 2017 CHFS covered 29 provinces (autonomous regions and municipalities directly under the Central Government), 353 counties (cities and districts), and 1417 communities (villages), with Hong Kong, Macao, Taiwan, Tibet, and Xinjiang as exceptions, and obtained microdata on a total of 40,011 households.

### 3.2. Variable Measurement

#### 3.2.1. Explained Variables

Based on Chaudhuri et al. [31], this research measures household poverty vulnerability from the perspective of vulnerability as expected poverty (VEP). Specifically, a three-stage feasible generalized least squares (FGLS) method is used to quantify the probability of future poverty for the sample households.

In the first step, we estimate the household consumption per capita equation [39], obtain the residual term, and then perform OLS estimation, as shown in Equation (1):

(1) $$\ln C_{i}=X_{i}\beta_{1}+e_{i}$$

where the subscripts of *i* = 1, 2, ……; N indexes the *ith* household; *lnC_i_* explains the logarithm of household consumption per capita; *X_i_* represents a series of consumption-related variables for household head and household characteristics; and *e_i_* represents the residual term.

In the second step, the squared consumption means and residuals are estimated by FGLS in a weighted regression, which in turn, yields the variance $\hat{\sigma }$*^2^_e,t_* and the expectation E of the logarithm of per capita consumption in the next period, as shown in Equations (2) and (3):

(2) $$E\left[\ln C_{i}|X_{i}\right]=X_{i}{\hat{\beta }}_{FGLS}$$

(3) $$\hat{V}\left[\ln C_{i}|X_{i}\right]={\hat{\sigma }}_{e,i}^{2}=X_{i}{\hat{\theta }}_{FGLS}$$

In Equation (2), *X_i_*$\hat{\beta }$*_FGLS_* is a consistent estimate of consumption expectation; in Equation (3), *X_i_*$\hat{\theta }$*_FGLS_* is a consistent estimate of consumption variance.

In the third step, household poverty vulnerability is calculated under the assumption that per capita household consumption in the next period follows a normal distribution, as shown in Equation (4):

(4) $${\hat{VEP}}_{i}=\hat{\mathrm{Pr}}\left(\ln C_{i}<\ln Z|X_{i}\right)=\Phi \left(\frac{\ln Z-X_{i}{\hat{\beta }}_{FGLS}}{\sqrt{X_{i}{\hat{\theta }}_{FGLS}}}\right)$$

where $\hat{VEP}$*_i_* is the poverty vulnerability of household *i*. Specifically, a household is considered to be vulnerable to poverty and takes a value of 1 when its probability of future poverty exceeds a set vulnerability line; otherwise, it takes a value of 0. In addition, ln*Z* is the logarithm of the poverty line.

This study uses the consumption poverty line developed by the World Bank. Precisely, USD 1.9 per day is mainly chosen as the main poverty criterion in this paper to calculate household poverty vulnerability, whereas the USD 3.1 poverty line is used for robustness testing. Based on the different poverty lines, this paper adopts the probability values of 29% and 50% as the thresholds of poverty vulnerability, based on the studies of Günther and Harttgen [32], to measure the vulnerability lines of low and high poverty vulnerability, respectively.

**Figure 1:**
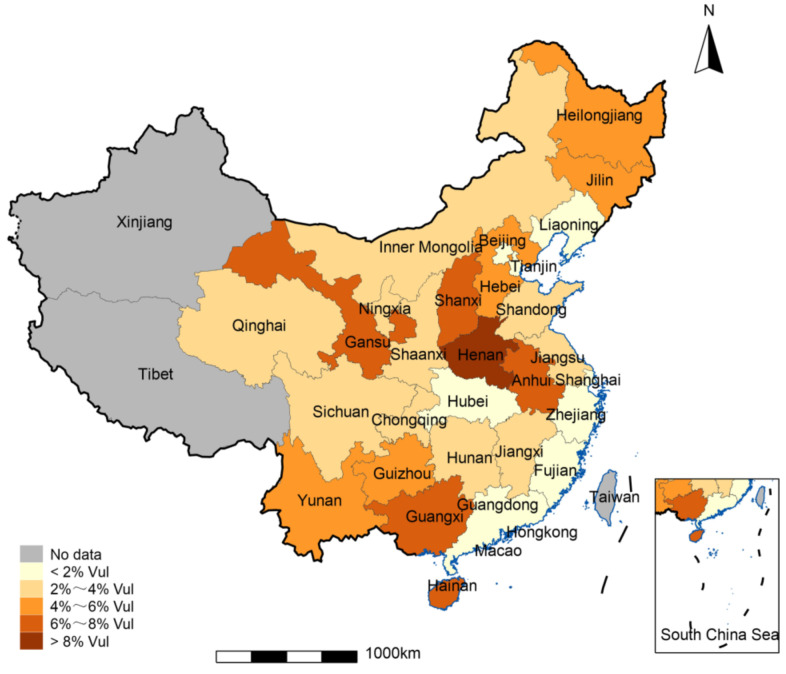
Probability of a household falling into poverty in the future by province (data source: 2017 CHFS).

To further reflect the probability of households falling into poverty in the future in different provinces, this study calculated the household poverty vulnerability (Vul) under the USD 1.9 poverty line based on the 2017 CHFS and mapped the geographical distribution of household poverty vulnerability in different provinces (see Figure 1). It can be found that excluding the regions in Xinjiang, Tibet, and the parts of Hong Kong, Macao, and Taiwan for which no data are available, there are different degrees of risk of returning to poverty across China. From the perspective of geographical location, the provinces with a probability of falling into poverty by more than 6% in the future are mainly located in the three eastern provinces of China, i.e., Gansu, Shanxi, Henan, Yunnan, Guangxi, and Hainan; the provinces with a probability of less than 2% are mainly distributed in China’s coastal areas, Beijing and Tianjin; and the probability of other provinces is basically between 2% and 6%, mainly located in the central and western regions of China. Overall, there is a relative concentration of household poverty vulnerability in different provinces in China, and there is obvious regional heterogeneity. These results suggest that if a long-term mechanism is not established to prevent poverty return and reduce vulnerability, there is a risk of a large-scale and concentrated poverty return to China.

#### 3.2.2. Core Explanatory Variable

The China Household Finance Questionnaire asks households about some of the payment methods (multiple choices possible) they typically use when shopping (including online shopping), such as cash; swiping a card (including bank cards, credit cards, etc.); paying through a computer (including internet banking, Alipay, etc.); paying through mobile terminals such as mobile phones and iPads (including Alipay App, WeChat Pay, mobile banking, Apple Pay, etc.); and others. In this paper, payment via mobile terminals such as mobile phones and iPads (including Alipay App, WeChat Pay, Mobile Banking, Apple Pay, etc.) is used to indicate that the household has used mobile payments and takes the value of 1; otherwise, it takes the value of 0 [63].

**Figure 2:**
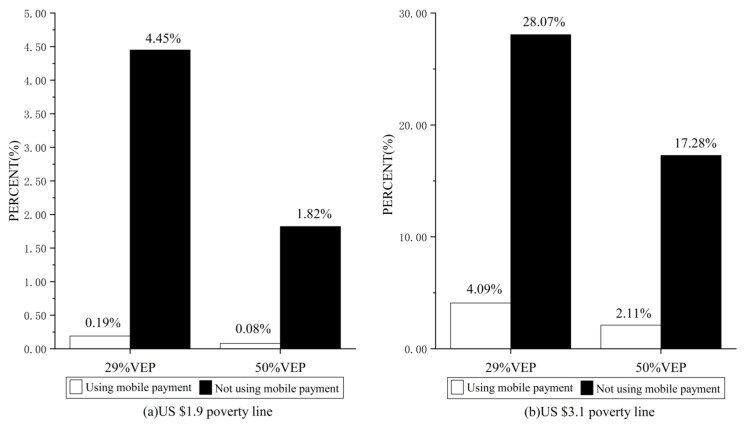
Descriptive statistics for different subgroups of household poverty vulnerability.

The results of the subgroup descriptive statistics are presented in Figure 2, which shows that the probability of being vulnerable to poverty at the USD 1.9 poverty line is 0.19% and 0.08% for households that make mobile payments, respectively, which are both much lower than for households that do not use mobile payments. In addition, mobile payments significantly reduce the probability of household poverty vulnerability at the USD 1.9 poverty line compared to the USD 3.1 poverty line.

#### 3.2.3. Other Control Variables

The control variables are the characteristic variables of the household head, the household characteristic variables, and the area dummy variables [61,62,64]. The household characteristic variables include gender of the household head, age, education level, risk preference, risk aversion, and if the household head is a farmer. The household characteristic variables are household size, household size squared, household debt, household transfer expenditure, number of unhealthy people in the household, household nonfinancial assets, and household home ownership; the regional dummy variable includes rural areas. Table 2 shows the main variable definitions.

### 3.3. Model, Variable Processing, and Description

Since VEP_1_ and VEP_2_ are both 0–1 variables, this study intends to use the probit model stepwise regression to test Hypothesis H1.

(5) $$\mathrm{Pr}\left(VEP_{i}=1|{\begin{array}{l} Mobile\;payment \end{array}}_{i},X_{i}\right)=\beta_{0}+\beta_{1}{\begin{array}{l} Mobile\;payment \end{array}}_{i}+\beta_{2}X_{i}+\varepsilon_{i}$$

where *VEP*_1_ = 1 indicates that household *i* is a poor and vulnerable household; otherwise, 0. *β*_0_ indicates the constant term. *β*_1_ and *β*_2_ indicate the coefficients of each variable. Mobile payment is the core explanatory variable in this model, which indicates a value of 1 if the household uses mobile payments; otherwise, 0. *X_i_* indicates the control variable. *ε_i_* is the random error term and $\varepsilon_{i}\sim N\left(0,\sigma^{2}\right)$. In addition, all regressions in this paper control for the fixed effect of provinces and adopt the robustness standard error clustered to the provincial level.

Following the needs of the methodology, this paper eliminates relevant missing variables referring to the existing literature, and finally, obtains 13,798 observations; the continuous variables such as transfer expenditure and household nonfinancial assets are winsorized by 0.5%. Furthermore, considering the effect of consumption heterogeneity between those who fail to age and those who are elderly heads of households, and concerning the Chinese retirement system (the retirement age in China is generally 60 for men and 55 for women), the paper limits the age of the head of household to between 18 and 65 years. Descriptive statistics for the main variables are shown in Table 3.

As can be seen from Table 3, the mean shares of households with low poverty vulnerability (VEP_1_) and high poverty vulnerability (VEP_2_) in the 2017 sample are about 2.8% and 1.2%, respectively, indicating that China’s poverty eradication efforts have been effective and the fruits of development have benefited all people. The proportion of households using mobile payments is only about 37.8%, indicating that the development of new digital financial models in China has only just begun and that mobile payment methods developed through new digital technologies, such as big data and the Internet, are far from universal.

### 3.4. Identifying Strategy and Estimation Method

Given the potential for omitted variables and reverse causality endogeneity in Equation (5), where the use of mobile payments is influenced by unobservable factors such as local culture and personal habits, and where households may choose to use mobile payments to collect money for profit to reduce their poverty vulnerability, the instrumental variables are tested. As the majority of current online purchases are made through mobile payments and there is no direct link between the presence or absence of online purchases and household poverty vulnerability, the correlation and homogeneity requirements of the instrumental variables are met. Given this, the basic regression is tested based on whether or not the household has experienced online shopping as an instrumental variable.

Since the explanatory and explained variables in this paper are both discrete variables, the two-stage least squares (2SLS) estimation results based on continuous variables may be biased; thus, a two-stage residual inclusion (2SRI) model is introduced for instrumental variable analysis.

Specifically, we use the 2SRI method to estimate the following two equations:

First stage:

(6) $$\;{\begin{array}{l} Mobile\;payment \end{array}}_{i}=\alpha_{0}+\alpha_{1}Online\;shopping_{i}+\alpha_{2}X_{1}{}_{i}$$

Second stage:

(7) $$\mathrm{Pr}\left(VEP_{i}=1\right)=\Phi \left(\beta_{0}+\beta_{1}{\begin{array}{l} Mobile\;payment \end{array}}_{i}+\beta_{2}X_{2i}+\beta_{3}{\hat{x}}_{ui}\right)+e^{2SRI}$$

Equation (6) is a linear regression in the first stage based on whether the household participates in online shopping and uses mobile payments. Equation (7) is the second stage of the probit regression based on the residuals from the first stage with household poverty vulnerability to control for endogeneity.

Here, online shopping is the instrumental variable and mobile payment is the endogenous variable. *X_i_* is the control variable, which mainly includes household characteristics, householder characteristics, and regional dummy variables. ${\hat{x}}_{ui}$ is the fitted value of residuals calculated from the first-stage regression equation and *e^2SRI^* is the random error term of the 2SRI model. *α*_0_ and *β*_0_ represent the constant terms. *α*_1_, *α*_2_, *β*_1_, *β*_2_, and *β*_3_ denote the coefficients of each variable.

## 4. Results

### 4.1. Baseline Analysis

The results of the regression between mobile payments and household poverty vulnerability at low and high vulnerability lines are presented in Table 4. In Table 4, columns (1) and (5) show the results of the test after controlling for province fixed effects only. The results both indicate that there is a significant negative relationship between the marginal effect of mobile payments on household vulnerability to poverty at the 1% level, suggesting that household use of mobile payments helps to reduce the probability of poverty vulnerability. Columns (2) and (6) control for the householder characteristic variable in columns (1) and (5), respectively, where the marginal effect of mobile payments increases but remains significantly negative at the 1% level. Columns (3) and (7) show the full estimation results. After controlling for the effects of other factors, the marginal effects of mobile payments on household poverty vulnerability at 29% and 50% probability values are −0.030 and −0.013, respectively, and both are statistically significant at the 1% level, supporting the inference of Hypothesis H1 and indicating that mobile payments have a greater impact on low vulnerability households (VEP_1_) than on high vulnerability households (VEP_2_). Among the control variables, the age of the householder, whether the householder is a farmer, and whether the household is in a rural area are positively associated with household poverty vulnerability; the years of education of the householder, household debt, household transfer expenditure, and household holdings of nonfinancial assets are negatively associated with household poverty vulnerability. The marginal effects of household size and the squared term of household size are opposite in sign and are significant at the 1% level, showing a clear inverted U-shaped relationship. This may be due to the increase in household size increasing the burden on the household, but if it is too large, it allows for economies of scale, which leads to an advantage of human capital over continuously depleted physical capital, ultimately alleviating household poverty vulnerability.

Columns (4) and (8) show the results of the second-stage regression of the instrumental variable online shopping with regard to low vulnerability to poverty (VEP_1_) and high vulnerability to poverty (VEP_2_), respectively. The results of Table 3 present a significant negative correlation between the two and the F-statistic of the one-stage regression is greater than the critical value at the 10% level, rejecting the hypothesis of a weak instrumental variable. In summary, it can be seen that the main findings of this study remain robust after controlling for endogeneity issues.

### 4.2. Robustness Check

The following robustness tests were conducted in this study (see Table 5): Firstly, the replacement of instrumental variables. This paper uses whether or not one owns a smartphone as the instrumental variable and, given the difficulty of assessing the validity of the instrumental variable in the 2SRI model, the 2SLS model is used as an auxiliary test. Columns (1) and (5) report the results of the second-stage regressions, where the marginal effect of the instrumental variable smartphone is significantly negative at least at the 5% level, and the results of the instrumental variable identifiability tests both reject the original hypothesis that the instrumental variable is not identifiable at the 1% level. The Cragg–Donald Wald F statistic also rejects the hypothesis of a weak instrumental variable. The Kleibergen–Paap rk LM statistic also rejected the original hypothesis of a weak instrumental variable, confirming that the instrumental variable was appropriate. In summary, the findings of this study still hold after the instrumental variable is replaced. Secondly, the replacement model. This study retested Hypothesis H1 using the logit model, and columns (2) and (6) report the results of their tests, both of which are consistent with the previous section, indicating that the conclusions of this study are robust. Third, vulnerability is estimated based on income. Referring to the studies by Zhang and Liu [65], poverty vulnerability is reconstructed using a poverty line defined in terms of income, and the results are presented in columns (3) and (7) of Table 5. The findings of the study remain unchanged after reconstructing poverty vulnerability with the income mean and income fluctuation models. Fourth, the poverty line was raised. The regression results in columns (4) and (8) show that the findings of the study still hold after the adoption of the USD 3.1 poverty line. The above robustness tests consistently show that household use of mobile payments mitigates poverty vulnerability, again validating Hypothesis H1.

### 4.3. Mechanism Analysis

#### 4.3.1. The Role of Entrepreneurial Decision-Making

When households fall into poverty vulnerability, they will be able to generate income and escape poverty by running businesses and industrial projects. The existing literature has provided relevant empirical evidence that mobile payments can facilitate undertaking productive activities such as entrepreneurship [63]. This study expands on this in two ways: Firstly, the sample is divided into two groups of farm and nonfarm households to discuss the differential impact of mobile payments on farm entrepreneurship. Second, dummy variables are set according to the survival of household entrepreneurship, drawing on the study by Song et al. [66]. If the household was engaged in commercial and industrial production at the time of the 2015 visit, but the original business ended at the time of the 2017 visit, the household is considered to have exited the business and is assigned a value of 1. Otherwise, it is assigned a value of 0. The opposite is true for the survival of a business. Households that were in production at the time of the 2017 visit are defined as starting a business and are assigned a value of 1; otherwise, they are assigned a value of 0.

This study divides the full sample into a two-part sample with different high and low types of vulnerability lines and uses the instrumental variable online shopping to test for transmission mechanisms; the regression results are presented in Table 6. Columns (1) and (2) indicate that the marginal effect of mobile payment is significantly positive at the 1% level in both the full sample and the rural sample, whereas the effect of mobile payments on entrepreneurship is not significant in the nonfarm household sample in column (3). Column (5) reports that entrepreneurship significantly reduces the probability of the occurrence of household poverty vulnerability at the high poverty vulnerability line, whereas column (4) indicates that this cannot occur at the low poverty vulnerability line. The regression results in column (6) suggest that household entrepreneurship survival is effective in improving poverty and that neither the entrepreneurial exit nor a start-up business contributes to reducing poverty vulnerability. These results suggest that mobile payments can have a positive effect on high poverty vulnerability groups by promoting entrepreneurship and significantly increasing the likelihood of entrepreneurial survival. In summary, Hypothesis H2 is validated.

#### 4.3.2. The Role of Risk Management Capabilities

The existing research suggests that household poverty vulnerability is influenced by the intensity of external risk shocks on the one hand, and internal risk management capacity on the other. In general, the stronger the external risk shock, the higher the probability that a household will fall into poverty, and if the household’s internal risk management capacity is poor, it is more likely to fall into poverty when faced with uncertain external risks. For this reason, mobile payments can provide households with effective financial services, such as insurance coverage or emergency loan support, to help them improve their risk management capacities to mitigate poverty vulnerability. In this study, the strength of a households’ risk management capacity is expressed in terms of “insurance coverage” and “cost of credit,” following the example of Urrea and Maldonado [67]. Specifically, households with unemployment insurance, commercial insurance, and access to credit at a lower cost indicate a higher risk management capacity.

The regression results are presented in Table 7, where columns (1), (2), and (3) show the regression results for the instrumental variable online shopping and the variables of unemployment insurance, business insurance, and cost of credit, respectively. The regression results for the variables of unemployment insurance, business insurance, and cost of credit show that mobile payments increase the probability of households purchasing insurance and increase the probability of obtaining a loan by reducing the cost of credit. Columns (4), (5), (7), and (8) show that at different levels of poverty vulnerability, household insurance coverage significantly reduces poverty vulnerability and the results are significant at the 1% level; columns (6) and (9) show the regression results for the cost of credit and low and high vulnerability, respectively. In summary, it can be seen that mobile payments can mitigate vulnerability through the channel of improving households’ risk management capacity, specifically in terms of both household insurance coverage and reduction in credit costs, thus testing Hypothesis H3.

### 4.4. Heterogeneity Analysis

#### 4.4.1. Population Differences

To answer the question of whether mobile payments are beneficial to the poor and vulnerable groups who traditionally do not have access to financial services and play a role in “financial inclusion,” this paper will further explore the role of mobile payments in poverty reduction in China, based on the perspective of household poverty vulnerability differences, both in terms of household income and whether the household owns a home.

Columns (1) and (5) in Table 8 report the moderating effect of mobile payments by household income for different low and high poverty vulnerabilities, respectively. The results show that the marginal effects of the interaction terms for both mobile payment and household income are significantly positive, at least at the 5% level, in mitigating different low and high poverty vulnerabilities, suggesting that mobile payments are more conducive to improving the poverty situation of low-income households. Columns (2) and (6), on the other hand, introduce an interaction term for mobile payment and housing ownership under the heterogeneous effect of low and high household vulnerability. The results show that mobile payments have a more mitigating effect on the poverty vulnerability of homeless households compared to housed households. This also implies that mobile payments play a role in the fight against poverty in China, supporting the sustainability of financial services for poor and vulnerable groups and driving the development of inclusive finance with digital finance.

#### 4.4.2. Regional Differences

Referring to existing studies, this study examines the poverty reduction effects of mobile payments in areas of weak economic and social development. Columns (3) and (7) and (4) and (8) in Table 8 show the heterogeneous effects of mobile payments on poverty vulnerability across regions and in rural and urban areas, respectively. As can be seen from columns (3) and (7) in Table 8, the marginal effects of the interaction term between mobile payments and the western region are all significantly negative, at least at the 5% level. With these results, it is suggested that mobile payments have a greater positive effect on household poverty vulnerability in the western region than in the other regions, using the eastern region as the reference group. The reason for this may be that the mitigating effect of mobile payments on poverty vulnerability is reduced in the eastern and central regions, which are rich in social resources and have a smaller base of poor people relative to the western region. Furthermore, as shown in columns (4) and (8) of Table 8, the marginal effect of the interaction term between mobile payments and rural areas is insignificant for low poverty vulnerability, but significantly negative for high poverty vulnerability, although this mitigating effect is weak. This may be due to the government’s use of modern information technology tools, such as mobile payments and transfer payments in the form of financial subsidies, to provide sustainable livelihoods for households in rural areas with high vulnerability.

## 5. Conclusions

In recent years, with the rapid development of China’s mobile payment methods and the significant achievements in the field of poverty alleviation, this paper combines the two to explore the relationship between mobile payments and household vulnerability to poverty, enriching the literature of ICT application. Based on the data of CHFS 2017, this study finds that: First, mobile payment is conducive to alleviating the vulnerability of households to poverty and has a more obvious effect on low poverty vulnerable households. Second, family entrepreneurship plays an intermediary role between mobile payment and poverty vulnerability. Among them, mobile payment is more conducive to promoting farmers’ entrepreneurship, and only the survival of entrepreneurship can significantly alleviate the vulnerability, whereas the impact of start-ups and entrepreneurial exits is not significant. Third, the risk management capability within the family plays an intermediary role between mobile payment and poverty vulnerability. Fourth, mobile payment can, to a certain extent, alleviate the negative impact on low-income and homeless families with poverty vulnerability. Finally, mobile payment plays a positive role in alleviating the poverty vulnerability of western regions and rural households.

Our research not only confirms that mobile payments can help reduce the probability of households falling into poverty in the future, but also validates the universality of mobile payments and their ability to bring benefits to vulnerable groups in China. This has important policy implications not only for China, but for other developing countries committed to the prevalence and development of mobile payments. For example, the “Digital China” strategy put forward by the Chinese government in recent years provides an external foundation for the popularization of mobile payment. At the same time, the Chinese government also takes into account the internal factors of vulnerable groups using mobile payment, and improves their digital literacy by formulating relevant public policies. This provides an important reference for the formulation of relevant information policies.

This paper makes relevant recommendations from both government and household perspectives for China. For the government: The government should actively promote the application of financial technology in poor areas, establish a preventive mechanism for households with different vulnerabilities aimed at preventing the slide from vulnerable poverty to poverty, and empower precise poverty alleviation and eradication. At the same time, financial institutions should be guided to provide sustainable financial supplies such as credit, investment, and insurance to households in less developed areas such as the west, especially in poor rural areas, and to continuously improve the entrepreneurial environment in conjunction with tax and fee reductions and other policy instruments to reduce the probability of households exiting entrepreneurship. For vulnerable households: They should make full use of external financial tools such as mobile payments to access the financial services needed; alleviate the difficulties and costs of financing for vulnerable households in starting their businesses; and enhance their risk management capabilities, such as risk prevention, risk avoidance, risk containment, and risk transfer. At the same time, they should also explore their internal motivation; make full use of the state’s one-off business start-up subsidy, guaranteed business start-up loans, entrepreneurial skills training, and other entrepreneurial policies to help the poor; become self-reliant; start their businesses; enhance the “blood generating” function; improve the quality of entrepreneurship; ensure the survival and development of entrepreneurship; and play a long-term role in poverty alleviation.

There are still limitations to this study: Firstly, due to data availability, only cross-sectional data are used in this study, although the VEP method can improve this situation to a certain extent. Secondly, the findings show that mobile payments can alleviate poverty by improving internal risk management capabilities, whereas the impact of external shocks and the prevention mechanisms need to be further explored. Thirdly, the role of mobile payments in public policies and benefits implemented by governments is worthy of a more in-depth study, as it may often be unprofitable for some households with high poverty vulnerability to be served by financial institutions.

## Figures and Tables

**Table 1:** Representative viewpoints on the relationship between mobile payment and poverty.

	Author, Year	Viewpoints	Critical Analysis
**First aspect**	Qiu et al., 2022 [47]	Mobile payment can increase rural household income and reduce the health risks of family members	The data need to be further improved
	Yu et al., 2022 [48]	Using mobile payment helps reduce COVID-19 infection	The impact of the external institutional environment on mobile payment remains to be discussed
	Suri et al., 2016 [49]	Mobile payment can lift households out of poverty and increase their consumption	The universality of mobile payment for vulnerable groups is not explained from the perspective of dynamic poverty
	Kikulwe et al., 2014 [50]	Mobile payment can help to overcome some of the important smallholder market access constraints that obstruct rural development and poverty reduction	The impact mechanism of mobile payment on informal savings and insurance needs to be explored
**Second aspect**	Sun et al., 2020 [51]	Credit can reduce vulnerability to poverty	The mechanism of credit channels on poverty vulnerability remains to be explored
	Wang et al., 2022 [52]	Digital finance can reduce poverty vulnerability	The influence mechanism between the two needs to be explored
	Zameer et al., 2020 [53]	Financial development positively contributes to poverty alleviation efficiency in China	Not considered from the perspective of dynamic poverty
	Scandurra et al., 2020 [54]	External funds can reduce the vulnerability of small island developing states	The estimation is biased because the political purpose of external funds is not taken into account

**Table 2:** Variable definition.

Variable	Definition
Mobile payment	Using mobile payment: 1, otherwise: 0
VEP_1_	USD 1.9 poverty line, 29% vulnerability line
VEP_2_	USD 1.9 poverty line, 50% vulnerability line
Age	Householder between the ages of 18 and 65
Gender	Householder gender; male: 1, female: 0
Education (years)	Householder education status; illiteracy: 0, primary school: 6, junior high school: 12, college/higher vocational school undergraduate college: 16, master’s degree: 19, doctoral degree: 22
Risk preference	Householder risk preference: 1, other: 0
Risk aversion	Householder risk aversion: 1, other: 0
Farmer	Householder is a farmer: 1, other: 0
Family size	Number of family members
Family size squared	The square of the number of family members
Debt	Household debt plus 1, then take the natural logarithm
Transfer expenditure	Household’s transfer expenditure plus 1, then take the natural logarithm
Unhealthy ^1^	Number of people in the household who rated themselves as unhealthy
Household nonfinancial assets	Household holdings of nonfinancial assets plus 1, then the natural logarithm
Housing ownership	Family owns its own home: 1, other: 0
Rural	Household is in a rural area: 1, other: 0

^1^ The question in the CHFS questionnaire measuring unhealthy people in the household is: How is the respondent’s current physical condition compared to his or her peers? The options for health status of the household members were very good; good; fair; bad; and very bad. In this paper, respondents who answered not good and very bad are defined as self-rated unhealthy.

**Table 3:** Variable descriptive statistics.

Variable	Observation	Mean	SD	Minimum	Maximum
Mobile payment	13,798	0.378	0.485	0	1
VEP_1_	13,798	0.028	0.166	0	1
VEP_2_	13,798	0.012	0.107	0	1
Age	13,798	48.42	10.20	18	65
Gender	13,798	0.839	0.368	0	1
Education	13,798	9.970	3.936	0	22
Risk preference	13,798	0.117	0.321	0	1
Risk aversion	13,798	0.574	0.495	0	1
Farmer	13,798	0.595	0.491	0	1
Family size	13,798	3.291	1.411	1	14
Family size squared	13,798	12.82	11.69	1	196
Debt	13,798	4.415	4.992	0	15.54
Transfer expenditure	13,798	0.474	0.507	0	4.879
Unhealthy	13,798	0.382	0.727	0	6
Household nonfinancial assets	13,798	12.69	1.764	5.303	16.36
Housing ownership	13,798	0.851	0.356	0	1
Rural	13,798	0.350	0.477	0	1

**Table 4:** Impact of mobile payment on household poverty vulnerability: the probit and 2SRI model.

	(1)	(2)	(3)	(4)	(5)	(6)	(7)	(8)
	VEP_1_				VEP_2_			
Mobile payment	−0.076 ***	−0.048 ***	−0.030 ***	−0.037 ***	−0.037 ***	−0.022 ***	−0.013 ***	−0.013 ***
	(0.007)	(0.007)	(0.005)	(0.006)	(0.004)	(0.004)	(0.003)	(0.005)
Gender		0.025 ***	0.016 ***	0.016 ***		0.008 **	0.005 *	0.006 *
		(0.006)	(0.005)	(0.005)		(0.004)	(0.003)	(0.003)
Age		0.001 ***	0.001 ***	0.001 ***		0.001 ***	0.001 ***	0.001 ***
		(0.000)	(0.000)	(0.000)		(0.000)	(0.000)	(0.000)
Education		−0.005 ***	−0.003 ***	−0.002 ***		−0.003 ***	−0.002 ***	−0.001 ***
		(0.001)	(0.000)	(0.000)		(0.000)	(0.000)	(0.000)
Risk preference		−0.012 ***	−0.002	−0.001		−0.003	0.005	0.006 *
		(0.004)	(0.003)	(0.003)		(0.003)	(0.003)	(0.003)
Risk aversion		−0.005	0.004 **	0.003 *		−0.002	0.003 **	0.003 **
		(0.003)	(0.002)	(0.002)		(0.002)	(0.002)	(0.002)
Farmer		0.047 ***	0.027 ***	0.024 ***		0.027 ***	0.019 ***	0.018 ***
		(0.007)	(0.006)	(0.006)		(0.006)	(0.006)	(0.005)
Family size			0.053 ***	0.054 ***			0.033 ***	0.032 ***
			(0.003)	(0.003)			(0.002)	(0.002)
Family size squared			−0.004 ***	−0.004 ***			−0.002 ***	−0.002 ***
			(0.000)	(0.000)			(0.000)	(0.000)
Housing ownership			0.066 ***	0.061 ***			0.034 ***	0.031 ***
			(0.008)	(0.008)			(0.006)	(0.006)
Rural			0.019 ***	0.016 ***			0.006 ***	0.004 ***
			(0.003)	(0.003)			(0.002)	(0.002)
Debt			−0.000 **	−0.000 *			−0.001 ***	−0.001 ***
			(0.000)	(0.000)			(0.000)	(0.000)
Transfer expenditure			−0.056 ***	−0.053 ***			−0.036 ***	−0.033 ***
			(0.004)	(0.004)			(0.006)	(0.006)
Unhealthy			−0.000	−0.001			−0.000	−0.000
			(0.001)	(0.001)			(0.001)	(0.001)
Household nonfinancial assets			−0.025 ***	−0.023 ***			−0.014 ***	−0.013 ***
			(0.001)	(0.001)			(0.000)	(0.000)
Province fixed effects	YES	YES	YES	YES	YES	YES	YES	YES
Observations	12,030	12,030	12,030	11,984	10,394	10,394	10,394	10,354
Adj. R^2^	0.140	0.235	0.666		0.127	0.231	0.742	
F test				426.33				426.33

Note: Standard errors are reported in parentheses. Data are from CHFS2017. *, **, and *** represent 10%, 5%, and 1% levels of statistical significance, respectively. The results in columns 4 and 8 are estimated by 2SRI, and the instrumental variable is whether the household member shops online or not. YES means to control for those factors. Marginal effects are reported in the table and, therefore, constant terms are not reported. The sample size in the baseline regression may not equal the overall sample size as the original data have varying degrees of missing values for different variables.

**Table 5:** Mobile payment on household poverty vulnerability: robustness check.

	(1)	(2)	(3)	(4)	(5)	(6)	(7)	(8)
	VEP_1_				VEP_2_			
Mobile payment	−0.160 ***	−0.028 ***	−0.050 ***	−0.032 ***	−0.074 **	−0.013 ***	−0.029 ***	−0.020 ***
	(0.051)	(0.005)	(0.007)	(0.005)	(0.031)	(0.003)	(0.007)	(0.004)
Controls	YES	YES	YES	YES	YES	YES	YES	YES
Province fixed effects	YES	YES	YES	YES	YES	YES	YES	YES
Observations	13,796	12,030	13,798	13,798	13,796	10,394	13,798	13,798
Adj. R^2^		0.669	0.563	0.742		0.746	0.489	0.747
Kleibergen–Paap rk LM Statistic	18.889				18.889			
Cragg–Donald Wald F statistic	157.721				157.721			
Hausman test	12.157 ***				6.600 **			
(*p*-value)	(0.002)				(0.016)			

Note: Standard errors are reported in parentheses. Data are from CHFS2017. **, and *** represent 5%, and 1% levels of statistical significance, respectively. The results in columns 1 and 4 are estimated by 2SLS and the instrumental variable is whether the household member uses a smartphone or not. YES means to control for those factors. Marginal effects are reported in the table and, therefore, constant terms are not reported. The sample size in the baseline regression may not equal the overall sample size as the original data have varying degrees of missing values for different variables.

**Table 6:** The results of the entrepreneurship mechanism test.

	(1)	(2)	(3)	(4)	(5)		(6)
	Entrepreneurship			VEP_1_	VEP_2_		Entrepreneurship Survival
Mobile payment	0.070 ***	0.117 ***	0.044				0.059 ***
	(0.023)	(0.026)	(0.031)				(0.016)
Entrepreneurship				−0.008	−0.009 **		
				(0.006)	(0.004)		
Entrepreneurship survival						−0.010 **	
						(0.004)	
Entrepreneurial exit						−0.003	
						(0.006)	
Start-up business						−0.002	
						(0.005)	
Controls	YES	YES	YES	YES	YES	YES	YES
Province fixed effects	YES	YES	YES	YES	YES	YES	YES
Observations	13,750	4806	8944	12,030	10,394	10,394	13,750
Adj. R^2^				0.658	0.740	0.740	
F test	426.33	25.28	267.42				426.33

Note: Standard errors are reported in parentheses. Data are from CHFS2017. **, and *** represent 5%, and 1% levels of statistical significance, respectively. The results in columns 1, 2, and 3 are estimated by 2SRI, and the instrumental variable is whether the household member shops online or not. YES means to control for those factors. Marginal effects are reported in the table and, therefore, constant terms are not reported. The sample size in the baseline regression may not equal the overall sample size as the original data have varying degrees of missing values for different variables.

**Table 7:** The results of the risk management capacity mechanism test.

	(1)	(2)	(3)	(4)	(5)	(6)	(7)	(8)	(9)
	Unemployment Insurance	Business Insurance	Cost of Credit	VEP_1_			VEP_2_		
Mobile payment	0.207 ***	0.146 ***	−0.037 **						
	(0.020)	(0.011)	(0.016)						
Unemployment insurance				−0.040 ***			−0.029 ***		
				(0.014)			(0.008)		
Business insurance					−0.027 ***			−0.016 ***	
					(0.010)			(0.006)	
Cost of credit						0.008 **			0.008 ***
						(0.004)			(0.002)
Controls	YES	YES	YES	YES	YES	YES	YES	YES	YES
Province fixed effects	YES	YES	YES	YES	YES	YES	YES	YES	YES
Observations	13,750	13,750	13,750	12,030	12,030	12,030	10,394	10,394	10,394
Adj. R^2^				0.424	0.423	0.422	0.445	0.452	0.455
F test	426.99	426.99	426.99						

Note: Standard errors are reported in parentheses. Data are from CHFS2017. **, and ***represent 5%, and 1% levels of statistical significance, respectively. The results in columns 1, 2, and 3 are estimated by 2SRI, and the instrumental variable is whether the household member shops online or not. YES means to control for those factors. Marginal effects are reported in the table and, therefore, constant terms are not reported. The sample size in the baseline regression may not equal the overall sample size as the original data have varying degrees of missing values for different variables.

**Table 8:** Results of the heterogeneity test.

	(1)	(2)	(3)	(4)	(5)	(6)	(7)	(8)
	VEP_1_				VEP_2_			
Mobile payment	−0.083 ***	−0.108 ***	−0.036 ***	−0.032 ***	−0.032 ***	−0.029 ***	−0.024 ***	−0.004
	(0.023)	(0.010)	(0.004)	(0.008)	(0.011)	(0.007)	(0.003)	(0.005)
West			−0.064 ***				−0.035 ***	
			(0.004)				(0.003)	
Mobile payment **×** west			−0.022 **				−0.056 ***	
			(0.009)				(0.005)	
Central			−0.017 ***				−0.006 ***	
			(0.003)				(0.002)	
Mobile payment **×** central			0.014				0.003	
			(0.009)				(0.005)	
Housing ownership		0.066 ***				0.034 ***		
		(0.008)				(0.006)		
Rural				0.019 ***				0.006 ***
				(0.003)				(0.002)
Mobile payment **×** household income	0.006 **				0.002 **			
	(0.003)				(0.001)			
Household income	−0.000				−0.000			
	(0.000)				(0.000)			
Mobile payment **×** housing ownership		0.078 ***				0.016 *		
		(0.009)				(0.009)		
Mobile payment **×** rural				0.004				−0.013 *
				(0.009)				(0.007)
Controls	YES	YES	YES	YES	YES	YES	YES	YES
Province fixed effects	YES	YES	YES	YES	YES	YES	YES	YES
Observations	12,030	12,030	12,030	12,030	10,394	10,394	10,394	10,394
Adj. R^2^	0.667	0.666	0.666	0.666	0.744	0.742	0.744	0.743

Note: Standard errors are reported in parentheses. Data are from CHFS2017. *, **, and *** represent 10%, 5%, and 1% levels of statistical significance, respectively. In our study, eastern China includes the provinces of Shanghai, Beijing, Tianjin, Shandong, Guangdong, Jiangsu, Hebei, Zhejiang, Hainan, Fujian, and Liaoning. Central and western China includes the provinces of Yunnan, Inner Mongolia, Jilin, Sichuan, Ningxia, Anhui, Shanxi, Guangxi, Jiangxi, Henan, Hubei, Hunan, Gansu, Guizhou, Chongqing, Shaanxi, Qinghai, and Heilongjiang. YES means to control for those factors. Marginal effects are reported in the table and, therefore, constant terms are not reported. The sample size in the baseline regression may not equal the overall sample size as the original data have varying degrees of missing values for different variables.

## Data Availability

Not applicable.

## References

[1] http://www.gov.cn/zhengce/2022-02/22/content_5675035.htm.

[2] http://rmfp.people.com.cn/n1/2020/0817/c406725-31824372.html.

[3] Jeanneney S.G., Kpodar K. (2011). Financial Development and Poverty Reduction: Can There Be a Benefit Without a Cost?. J. Dev. Stud..

[4] Beck T., Pamuk H., Ramrattan R., Uras B.R. (2018). Payment Instruments, Finance and Development. J. Dev. Econ..

[5] Korenman S.D. (2016). Including Health Insurance in Poverty Measurement: The Impact of Massachusetts Health Reform on Poverty. J. Health Econ..

[6] Pega F., Gilsanz P., Kawachi I., Wilson N., Blakely T. (2017). Cumulative Receipt of an Anti-Poverty Tax Credit for Families Did Not Impact Tobacco Smoking among Parents. Soc. Sci. Med..

[7] Visser M., Jumare H., Brick K. (2020). Risk Preferences and Poverty Traps in the Uptake of Credit and Insurance amongst Small-Scale Farmers in South Africa. J. Econ. Behav. Organ..

[8] Fu L., Li J., Fang X., Wei H. (2021). The Mechanism and Validation of Digital Inclusive Finance Promoting Inclusive Growth. Stat. Res..

[9] Akhter S., Daly K.J. (2009). Finance and Poverty: Evidence from Fixed Effect Vector Decomposition. Emerg. Mark. Rev..

[10] Dong K., Taghizadeh-Hesary F., Zhao J. (2022). How Inclusive Financial Development Eradicates Energy Poverty in China? The Role of Technological Innovation. Energy Econ..

[11] Xu L., Zhang Q., Shi X. (2019). Stakeholders Strategies in Poverty Alleviation and Clean Energy Access: A Case Study of China’s PV Poverty Alleviation Program. Energy Policy.

[12] Blau B.M. (2018). Income Inequality, Poverty, and the Liquidity of Stock Markets. J. Dev. Econ..

[13] Pradhan R.P., Arvin M.B., Nair M.S., Hall J.H., Bennett S.E. (2021). Sustainable Economic Development in India: The Dynamics between Financial Inclusion, ICT Development, and Economic Growth. Technol. Forecast. Soc. Chang..

[14] Agyekum F.K., Reddy K., Wallace D., Wellalage N.H. (2022). Does Technological Inclusion Promote Financial Inclusion among SMEs? Evidence from South-East Asian (SEA) Countries. Glob. Financ. J..

[15] Hewa Wellalage N., Hunjra A.I., Manita R., Locke S.M. (2021). Information Communication Technology and Financial Inclusion of Innovative Entrepreneurs. Technol. Forecast. Soc. Chang..

[16] Cao S., Nie L., Sun H., Sun W., Taghizadeh-Hesary F. (2021). Digital Finance, Green Technological Innovation and Energy-Environmental Performance: Evidence from China’s Regional Economies. J. Clean. Prod..

[17] Wang H., Guo J. (2022). Impacts of Digital Inclusive Finance on CO2 Emissions from a Spatial Perspective: Evidence from 272 Cities in China. J. Clean. Prod..

[18] Yuan S., Musibau H.O., Genç S.Y., Shaheen R., Ameen A., Tan Z. (2021). Digitalization of Economy Is the Key Factor behind Fourth Industrial Revolution: How G7 Countries Are Overcoming with the Financing Issues?. Technol. Forecast. Soc. Chang..

[19] Thompson B.S. (2017). Can Financial Technology Innovate Benefit Distribution in Payments for Ecosystem Services and REDD+?. Ecol. Econ..

[20] Dixon P.N., Fox C.A., Kelley E.K. (2021). To Own or Not to Own: Stock Loans around Dividend Payments. J. Financ. Econ..

[21] Verkijika S.F. (2020). An Affective Response Model for Understanding the Acceptance of Mobile Payment Systems. Electron. Commer. Res. Appl..

[22] Liu Y., Yan W., Hu B. (2021). Resistance to Facial Recognition Payment in China: The Influence of Privacy-Related Factors. Telecommun. Policy.

[23] Koomson I., Bukari C., Villano R.A. (2021). Mobile Money Adoption and Response to Idiosyncratic Shocks: Empirics from Five Selected Countries in Sub-Saharan Africa. Technol. Forecast. Soc. Chang..

[24] Rostad W.L., Klevens J., Ports K.A., Ford D.C. (2020). Impact of the United States Federal Child Tax Credit on Childhood Injuries and Behavior Problems. Child. Youth Serv. Rev..

[25] Sykes J., Križ K., Edin K., Halpern-Meekin S. (2015). Dignity and Dreams: What the Earned Income Tax Credit (EITC) Means to Low-Income Families. Am. Sociol. Rev..

[26] Wisner B., Blaikie P., Cannon T., Davis I. (1994). AT RISK: Natural Hazards, People’s Vulnerability and Disasters.

[27] Chan N.W., Aldrich D.P., Oum S., Sawada Y. (2015). Impacts of Disasters and Disaster Risk Management in Malaysia: The Case of Floods. Resilience and Recovery in Asian Disasters.

[28] Janssen M.A., Schoon M.L., Ke W., Börner K. (2006). Scholarly Networks on Resilience, Vulnerability and Adaptation within the Human Dimensions of Global Environmental Change. Glob. Environ. Chang..

[29] Liebow E.B. (2005). At Risk: Natural Hazards, People’s Vulnerability, and Disasters. J. Homel. Secur. Emerg. Manag..

[30] Zhang H., Han X. (2021). Sustainable Poverty Reduction Effect of Digital Finance: A Perspective of Household Poverty Vulnerability. Chin. Rev. Financ. Stud..

[31] Chaudhuri S. (2002). Assessing Household Vulnerability to Poverty from Cross-Sectional Data: A Methodology and Estimates from Indonesia.

[32] Günther I., Harttgen K. (2009). Estimating Households Vulnerability to Idiosyncratic and Covariate Shocks: A Novel Method Applied in Madagascar. World Dev..

[33] Jiang L. (2017). New progress in poverty vulnerability theory and policy research. Econ. Perspect..

[34] Magrini E., Montalbano P., Winters L.A. (2018). Households’ Vulnerability from Trade in Vietnam. World Dev..

[35] Mayadunne S., Park S. (2016). An Economic Model to Evaluate Information Security Investment of Risk-Taking Small and Medium Enterprises. Int. J. Prod. Econ..

[36] Azeem M.M., Mugera A.W., Schilizzi S., Siddique K.H.M. (2017). An Assessment of Vulnerability to Poverty in Punjab, Pakistan: Subjective Choices of Poverty Indicators. Soc. Indic. Res..

[37] Senapati A.K. (2020). Assessing the Vulnerability of Agricultural Households to Covariate and Idiosyncratic Shocks: A Case Study in Odisha, India. Clim. Dev..

[38] Yin Z., Zhang D. (2020). Financial Inclusion, Household Poverty and Vulnerability. China Econ. Q..

[39] Celidoni M. (2013). Vulnerability to Poverty: An Empirical Comparison of Alternative Measures. Appl. Econ..

[40] Chiwaula L.S., Witt R., Waibel H. (2011). An Asset-Based Approach to Vulnerability: The Case of Small-Scale Fishing Areas in Cameroon and Nigeria. J. Dev. Stud..

[41] Liu Z., Zheng W., Jia R., Jing P. (2019). Health Insurance, Health Heterogeneity, and Targeted Poverty Reduction: A Vulnerability to Poverty Approach. J. Financ. Res..

[42] Fan Z., Zhou W. (2021). From Anti-poverty Campaign to Common Prosperity: Dynamic Identification of Relative Poverty and Quantitative Decomposition of Poverty Changes in China. China Ind. Econ..

[43] Alemu B.T., Singh S.P. (2021). How Does Multidimensional Rural Poverty Vary across Agro-Ecologies in Rural Ethiopia? Evidence from the Three Districts. J. Poverty.

[44] Pritchett L., Suryahadi A., Sumarto S. (2000). Quantifying Vulnerability to Poverty: A Proposed Measure, with Application to Indonesia.

[45] Klasen S., Waibel H. (2015). Vulnerability to Poverty in South-East Asia: Drivers, Measurement, Responses, and Policy Issues. World Dev..

[46] Si L. (2019). Does Family Education Expenditure Reduce the Household’s Poverty Vulnerability in Rural China? An Empirical Study Based on CFPS Data. J. Financ. Econ..

[47] Qiu W., Wu T., Xue P. (2022). Can Mobile Payment Increase Household Income and Mitigate the Lower Income Condition Caused by Health Risks? Evidence from Rural China. Int. J. Environ. Res. Public Health.

[48] Yu S.-Y., Chen D.C. (2022). Consumers’ Switching from Cash to Mobile Payment under the Fear of COVID-19 in Taiwan. Sustainability.

[49] Suri T., Jack W. (2016). The Long-Run Poverty and Gender Impacts of Mobile Money. Science.

[50] Kikulwe E.M., Fischer E., Qaim M. (2014). Mobile Money, Smallholder Farmers, and Household Welfare in Kenya. PLoS ONE.

[51] Sun H., Li X., Li W. (2020). The Nexus between Credit Channels and Farm Household Vulnerability to Poverty: Evidence from Rural China. Sustainability.

[52] Wang X., Fu Y. (2022). Digital Financial Inclusion and Vulnerability to Poverty: Evidence from Chinese Rural Households. CAER.

[53] Zameer H., Shahbaz M., Vo X.V. (2020). Reinforcing Poverty Alleviation Efficiency through Technological Innovation, Globalization, and Financial Development. Technol. Forecast. Soc. Chang..

[54] Scandurra G., Thomas A., Passaro R., Bencini J., Carfora A. (2020). Does Climate Finance Reduce Vulnerability in Small Island Developing States? An Empirical Investigation. J. Clean. Prod..

[55] Lin Y., Shen Y., Sun A. (2020). Evaluating the Stimulus Effect of Consumption Vouchers in China. Econ. Res. J..

[56] Zhang X., Yang T., Wang C., Wan G. (2020). Digital Finance and Household Consumption: Theory and Evidence from China. Manag. World.

[57] Sutter C., Bruton G.D., Chen J. (2019). Entrepreneurship as a Solution to Extreme Poverty: A Review and Future Research Directions. J. Bus. Ventur..

[58] Williams C.C., Martinez–Perez A., Kedir A.M. (2017). Informal Entrepreneurship in Developing Economies: The Impacts of Starting up Unregistered on Firm Performance. Entrep. Theory Pract..

[59] Wang Y., Kan B. (2021). On the Effect of Internet Finance on Profitability of Commercial Banks. Financ. Econ..

[60] Wang X. (2020). Mobile Payment and Informal Business: Evidence from China’s Household Panel Data. China World Econ..

[61] Zhang J., Zhang H., Gong X. (2022). Mobile Payment and Rural Household Consumption: Evidence from China. Telecommun. Policy.

[62] Zhao C., Wu Y., Guo J. (2022). Mobile Payment and Chinese Rural Household Consumption. China Econ. Rev..

[63] Yin Z., Gong X., Guo P. (2019). The Impact of Mobile Payment on Entrepreneurship Micro Evidence from China Household Finance Survey. China Ind. Econ..

[64] Gan L., Yin Z., Jia N., Xu S., Ma S., Zheng L. (2014). Data You Need to Know about China.

[65] Zhang D., Liu W. (2022). Research on the Alleviation Effect of “Single-person Households” Enrollment on the Poverty Vulnerability of Low-income Groups-An Empirical Analysis Based on CFPS Data. Inq. Econ. Issues.

[66] Song Q., Wu Y., Yin Z. (2020). Impact of financial literacy on the survival of entrepreneurship. Sci. Res. Manag..

[67] Urrea M.A., Maldonado J.H. (2011). Vulnerability and Risk Management: The Importance of Financial Inclusion for Beneficiaries of Conditional Transfers in Colombia. Can. J. Dev. Stud./Rev. Can. D’études Du Développement.

